# Natural Crystal Structure for Generating Raman‐Like Orbital Angular Momentum States

**DOI:** 10.1002/advs.202500377

**Published:** 2025-04-25

**Authors:** Tianxiang Meng, Changsheng Zheng, Yongguang Zhao, Haohai Yu, Huaijin Zhang

**Affiliations:** ^1^ State Key Laboratory of Crystal Materials and Institute of Crystal Materials Shandong University Jinan 250100 China

**Keywords:** handedness selection, natural of crystal, orbital angular momentum of photon, Raman‐like OAM states, thermal‐driven mode competition

## Abstract

Creation of structured light at‐source with determinate orbital angular momentum (OAM) states is a fascinating branch of modern optics, owing to their integrability, wavelength insensitivity, and high mode purity. However, mode degeneracy in spatially column‐symmetric cylindrical coordinate systems leads to a total zero OAM states which seriously limits their development and applications. Here, a strategy is proposed for symmetry breaking of the degenerate OAM states relying on the natural anisotropy of crystal gain, to select the handedness of the spatial spiral phase front and further tunable Raman‐like OAM states by thermal‐driven, experimentally realized arbitrary OAM states from −2*ћ* to *ћ*. The concept presented herein clarifies the contribution of intrinsic crystal property to the OAM states and opens a new route for an at‐source solution of structure light with high integrability and controllable OAM states.

## Graphical abstract

This work proposes a strategy for symmetry breaking of the degenerate OAM states relying on the natural anisotropy of crystal gain, to select the handedness of the spatial spiral phase front and further tunable Raman‐like OAM states by thermal‐driven, which clarifies the contribution of intrinsic crystal property to the OAM states and opens a new route for an at‐source solution of structure light with high integrability and controllable OAM states.

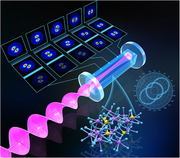

## 1 Introduction

As early as the 1900s, Poynting^[^ ^1^ ^]^ predicted that circularly polarized light could carry spin angular momentum (SAM), which was later confirmed by Richard Beth^[^ ^2^ ^]^ through changing the polarization state of light. In terms of the optical orbital angular momentum (OAM), however, no studies before Allen et al. in 1992^[^ ^3^ ^]^ recognized that optical vortices with spiral wavefront enable to give rise to a well‐defined OAM of *lћ* (*l*: topological charge which can be any integer) per photon. Since then, structure light carrying OAM has been widely studied due to their numerous potential applications,^[^ ^4^, ^5^, ^6^ ^]^ e.g., mode division multiplexing (MDM) in optical communications based on infinite OAM extends the transmission rates exceeding T‐bit levels,^[^ ^7^, ^8^ ^]^ the new degree of freedom could be exploited to create the 18‐qubit hyperentanglement of six photons in quantum information processing.^[^ ^9^ ^]^ In light‐matter interactions, the OAM light can trap and bind particles at the central singularity, and then driving the particle movement by the transform of optical OAM and mechanical angular momentum.^[^ ^10^, ^11^, ^12^ ^]^ In addition, based on its special light field, OAM light are also widely used in material processing, such as deep hole drilling,^[^ ^13^ ^]^ photopolymerization,^[^ ^14^, ^15^ ^]^ and nanopatterning,^[^ ^16^, ^17^ ^]^ especially with significant advantages in the processing of chiral materials.^[^ ^18^, ^19^, ^20^ ^]^

The production of OAM modes relied on the conversion of pre‐existing beams by modulation elements in early days, e.g., the Hermite‐Gaussian (HG) mode is converted into a Laguerre‐Gaussian (LG) mode by the introduction of an additional Gouy‐phase difference (±π/2),^[^ ^21^ ^]^ transforming the fundamental mode into a vortex beam by imposing a helical phase (2π*l*) or transform circularly polarized light into OAM light via spin‐orbit coupling.^[^ ^22^ ^]^ OAM states directly generated in cavity are currently attracting increasing attention owing to their robust structure and ideal quality. Tailored laser modes can be achieved by inserting a phase modulation element such as a spatial light modulator (SLM), a *q*‐plate, or a metasurface into the cavity, or from the Janus cavity with a dual‐faced transverse mode structures.^[^ ^23^ ^]^ Digital lasers demonstrate real‐time switching between spatial modes in an otherwise standard solid‐state laser resonator,^[^ ^24^ ^]^ however, operating wavelength regions and power‐handling capability are limited by the damage of these phase elements. Another approach is to filter the desired OAM modes by mode selection in the cavity, e.g., amplitude tailoring including ring beam pumping^[^ ^25^, ^26^ ^]^ and intracavity point defects,^[^ ^27^ ^]^ or using strong spherical aberration^[^ ^28^ ^]^ to distinguish the optical paths of different modes to achieve a wide range of OAM tuning. Optical vortices with topological charges up to the 288th order are generated by laser inscribing round patterns on the surface of the cavity mirror,^[^ ^29^ ^]^ the angular modes of the output beam could be selected in the range of over 100 order.^[^ ^30^ ^]^ However, the produced laser modes are the degeneracy of azimuthal modes (± *l*) due to the symmetry of the cylindrical coordinate cavity, thereby additional symmetry breaking to select a well‐defined helicity is essential.^[^ ^31^ ^]^ Despite many attempts that have been used to break the helicity degeneracy, a simple and robust method is still lacking. The handedness controllable LG_0,1_ mode is obtained via the combination of HG_1,0_ and HG_0,1_ modes in the sagittal and tangential plane generation by off‐axis pumping, however, it relies on stabilized Gouy‐phase difference.^[^ ^32^ ^]^ A novel method was proposed and experimentally demonstrated to control the vortex helicity using a simple quarter‐wave plate, but the polarization dependence is not universal in OAM lasers.^[^ ^33^ ^]^ It is feasible to generate different transmission losses for each helical by misaligning the output coupler and inserting a tilted etalon or metal wires in the cavity, but this scheme requires precise manipulation.^[^ ^34^, ^35^, ^36^ ^]^ The effect of the natural gain medium on the optical vortices generation has long been neglected, to the detriment of resolving the evolution of the vortex beam inside the cavity and the interaction of light, materials, and the OAM.

Here, we provide a new method for the generation and handedness selection of OAM state lasers by using the natural properties of gain crystals, which enables the efficient and stable generation of well‐defined OAM state lasers without complex devices and sophisticated manipulation thereby avoiding insertion and asymmetric losses due to modulation elements. Experimentally realized arbitrary OAM states from −2*ћ* to *ћ* by thermal‐driven. We illustrate the origin of photonic OAM in low symmetry biaxial crystals and develop a simple model to understand the link between crystal anisotropy and handedness selection, then we analyze the dynamics of thermal‐induced mode tuning and provide guidelines for the output of high‐purity single‐handedness OAM state lasers. This simple method provides a possibility for the generation of structured light fields and opens up research ideas for the interaction between OAM and crystals.

## 2 Results

### 2.1 Theoretical Analysis of Laguerre‐Gaussian Complete Basis in Biaxial Crystal

The transverse modes of laser can be considered as structured 2D quantum wavepackets which are the operators of Helmholtz eigenfunctions,^[^ ^37^, ^38^ ^]^ called Helmholtz‐Gauss beams, such as Hermite‐Gaussian beams in the Cartesian coordinate, Ince‐Gaussian beams in the elliptic coordinates, and Laguerre‐Gaussian beams in cylindrical coordinates— a complete basis of OAM states. Apart from the artificial design, the low symmetry biaxial crystal itself enables to provision of a cylindrical‐coordinate system for the resonant modes in a laser cavity, as well as offering conical refraction and anisotropic gain. As passing through the biaxial crystal along its optical axes, the collimated beam is transformed first into a hollow oblique cone inside the crystal and then into a hollow optical cylinder outside the crystal, with the interaction between the angular momentums (both spin and orbital) of the photon and the crystal simultaneously,^[^ ^39^, ^40^ ^]^ e.g., for the input LG*_0,l_* beam which light field can be expressed as $E=E_{0}{\left(\frac{r\sqrt{2}}{\omega }\right)}^{\left|l\right|}\exp\left(-\frac{r^{2}}{\omega^{2}}\right)\exp\left(il\varphi \right)$, the output light field can be written as:^[^ ^41^ ^]^

(1) $$E_{\left(r\right)}^{\pm }=B_{l\left(r\right)}e^{il\theta }e^{\pm }+B_{l\pm 1\left(r\right)}e^{i\left(l\pm 1\right)\theta }e^{\mp }$$

where the ± denotes the left (right) circular polarization and the *B_l_* (*B_l+1_*) is labeled according to the order of the corresponding Bessel functions, indicating the amplitude of the corresponding component. The change in angular momentum of light results in a torque on the crystal,^[^ ^42^ ^]^ along the axis of the incident beam which is similar to the cylindrical lens mode converter. Consequently, the conical refraction of biaxial crystals leads to an OAM state component in the fundamental mode oscillation, which can be amplified as a seed by the stimulated radiation when this component overlaps with the pump light and generates the OAM state laser.

In addition, since the three main values of the permittivity *ε* in both the real and imaginary parts are different, it also exhibits spatially angular distribution of the refractive index, absorption, and fluorescence. Meanwhile, the absorption and fluorescence principal values of monoclinic crystals are not along the principal axis of the dielectric frame, but the principal axis of two new frames tilted from the dielectric frame.^[^ ^43^, ^44^ ^]^ The imaginary relative dielectric permittivity tensor can be diagonalized by a rotation matrix and introducing a specific principal frame(*X’, Y’, Z’*) called the absorption or fluorescence frame:

(2) $$\varepsilon =\left[\begin{array}{ccc} \varepsilon_{xx} & 0 & 0\\ 0 & \varepsilon_{yy} & 0\\ 0 & 0 & \varepsilon_{zz} \end{array}\right]+j\left[\begin{array}{ccc} \cos\alpha & 0 & \sin\alpha \\ 0 & 1 & 0\\ -\sin\alpha & 0 & \cos\alpha \end{array}\right]\left[\begin{array}{ccc} {\varepsilon^{\prime }}_{x^{\prime }x^{\prime }} & 0 & 0\\ 0 & {\varepsilon^{\prime }}_{y^{\prime }y^{\prime }} & 0\\ 0 & 0 & {\varepsilon^{\prime }}_{z^{\prime }z^{\prime }} \end{array}\right]$$

**Figure 1:**
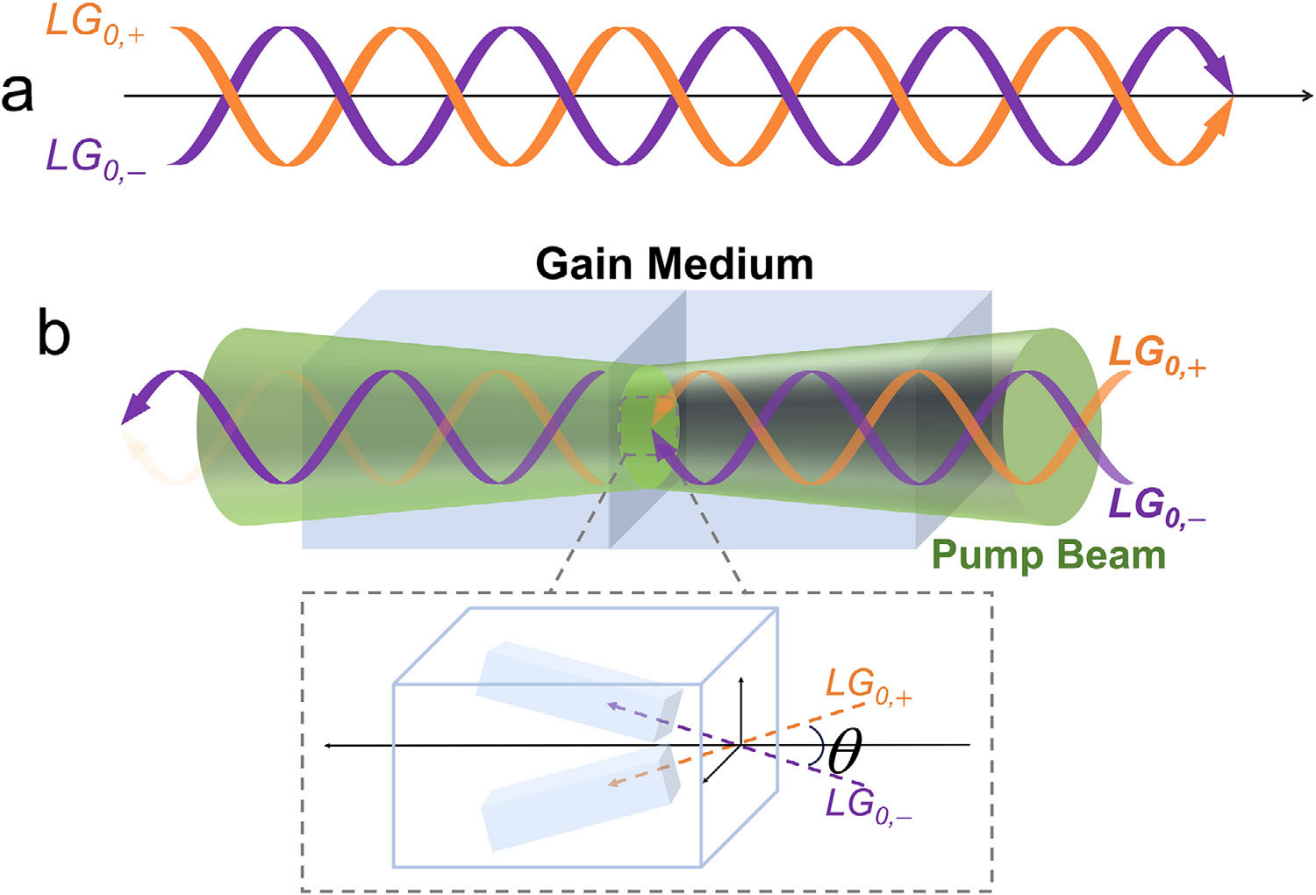
Handedness selection model based on natural gain anisotropy of the crystal. a) Propagation of Poynting vector of two optical vortices with opposite handedness in a helical pattern. b) Handedness selection of optical vortices in the crystal, where OAM states of opposite handedness at the focal plane of the pump beam acquire an anisotropic gain due to the different orientations of the Poynting vectors (illustrated at the bottom of the picture), and this disparity in intensity is amplified in the mode competition, ultimately leading to the disappearance of one handedness.

In cylindrical coordinate cavities, the simultaneous operation of the two handedness OAM modes leads to a petal‐like pattern of the coherent superposition of the laser field, resulting in the loss of its helical wavefront and OAM properties. The Poynting vector of photons carrying orbital angular momentum is helical despite the light field propagating in the direction of the optical axis (see **Figure** **1**a). The Poynting vector for a linearly polarized LG mode optical vortex beam can be expressed as:^[^ ^45^, ^46^ ^]^

(3) $$\vec{S}=\varepsilon_{0}\left\{\frac{\omega krz}{z_{R}^{2}+z^{2}}\hat{r}+\frac{\omega l}{r}\hat{\phi }+\omega k\hat{z}\right\}{\left|u\right|}^{2}$$

where *ε*_0_ is the vacuum dielectric constant, *ω* is the angular frequency, *r* and *ϕ* are the radial and azimuthal coordinates, *l* is the topological charge, *k* is the wavenumber, and *u* is the amplification. The angle of deviation of the vortex photon propagation direction from the optical axis can be expressed as:

(4) $$\theta =\frac{p_{\phi }}{p_{z}}=\frac{l\lambda }{2\pi r}$$

where *P_ϕ_* is the angular component of the Poynting vector and *λ* is the wavelength. It can be seen that the angle of deviation is related to the OAM. The low symmetry biaxial crystals display an anisotropic gain angular distribution as a consequence of their low‐symmetry structure,^[^ ^47^ ^]^ which implies that light traversing the crystal in disparate directions exhibits disparate gains. Consequently, the two handedness OAM states with disparate Poynting vectors exhibits intensity disparities attributable to the varying gains attained in the crystal. This removal of degeneracy does not break the symmetry of the cavity, thereby averting any loss of efficiency (see Figure 1b). Therefore, we believe that monoclinic crystals are the ideal gain medium for directly generated OAM lasers.

### 2.2 Experimental Demonstration of Well‐Defined and Tunable OAM States

**Figure 2:**
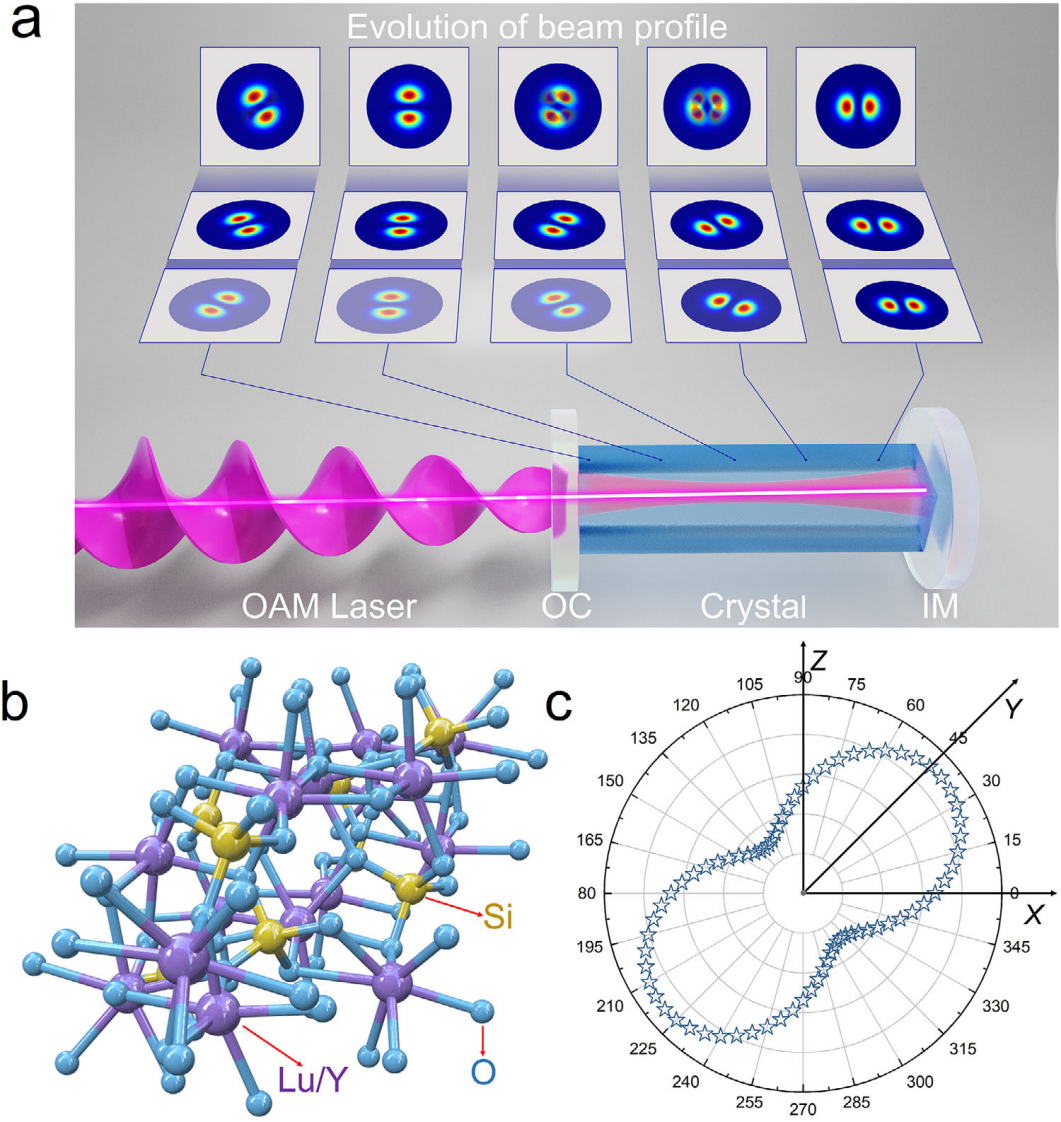
Experimental demonstration of OAM laser with Nd:LYSO crystal. a) Schematic of the OAM laser using a Fabry‐Perot cavity, where the top picture shows the mode evolution and intensity patterns evolution in the cavity. b) The structure of monoclinic Nd:LYSO crystal with C2/c space group. c) Stimulated emission cross section of the *Y*‐cut Nd:LYSO crystal, where the E//X polarized beam has an anisotropic emission due to the low symmetry structure.

The OAM laser schematic is illustrated in **Figure** **2**a, a monoclinic Nd:LYSO crystal was selected as the gain medium (see Methods) for OAM laser operation. As a biaxial crystal, the Nd:LYSO crystal exhibits the advantages of large volume, good thermal properties, anisotropic stimulated emission cross sections, and pronounced polarization properties due to its low symmetry structure (see Figure 2b). For *Y*‐cut crystals, the stimulated emission cross section of *E ∥ X*‐polarized light can be expressed as in the X‐Z plane:^[^ ^48^ ^]^

(5) $$\sigma_{XZ}\left(\theta \right)=\sigma_{X}\cos^{2}\left(\theta \right)+\sigma_{Z}\sin^{2}\left(\theta \right)+{\sigma^{\prime }}_{XZ}\cos\left(\theta \right)\sin\left(\theta \right)$$

as shown in Figure 2c, where *σ_X_* is the stimulated emission cross section of *E ∥ X* polarized, while *σ_Z_* is the stimulated emission cross section of *E ∥ Z* polarized.

**Figure 3:**
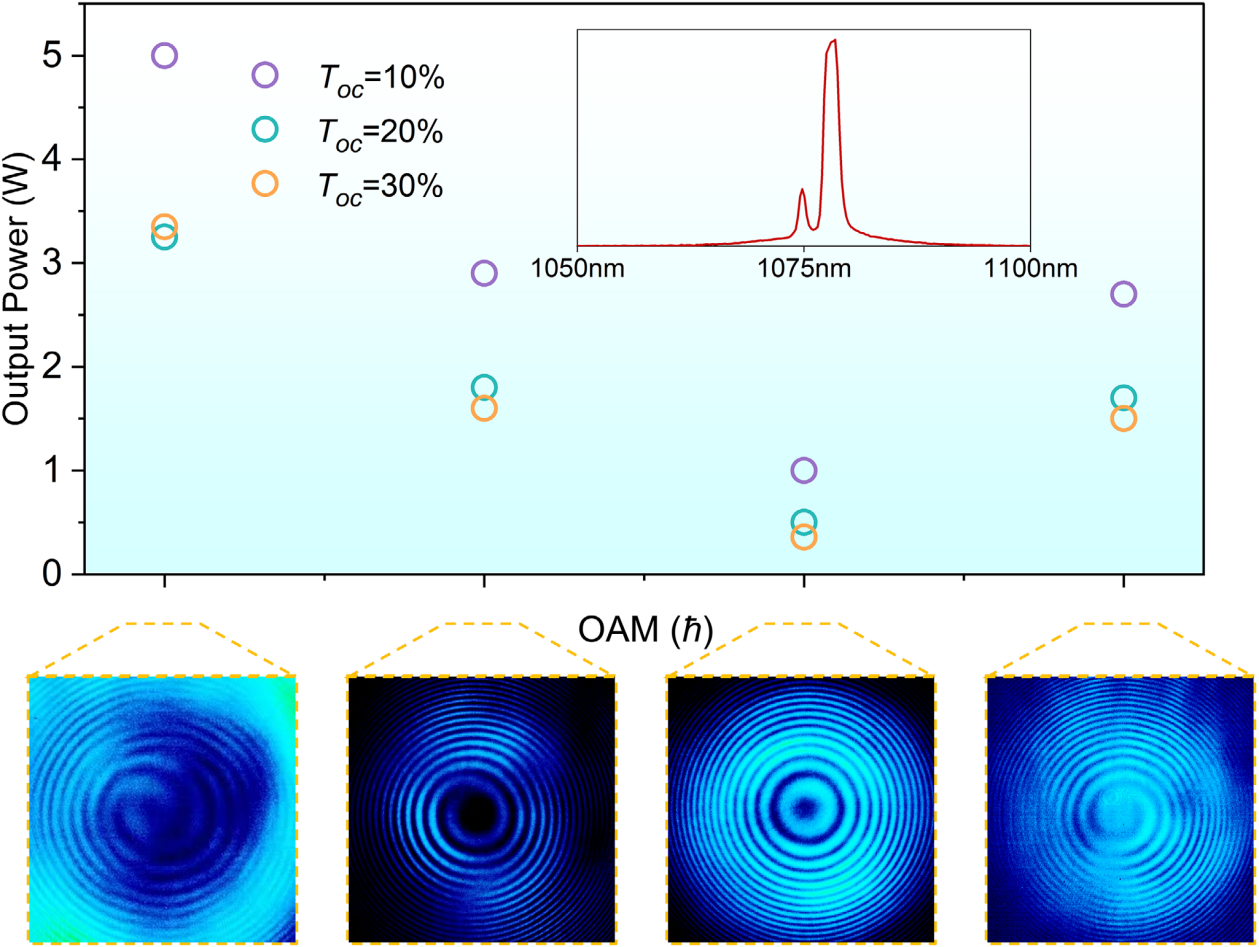
Laser performance of OAM states and the corresponding interferograms. The figure shows the power handling capacity of the output coupler for three different transmittances, with the maximum power obtained by the output coupler transmittance is 10%. The center wavelength of the laser is 1078 nm, the maximum power of LG_0,‒2_ mode is 5.05 W, the power of LG_0,‒1_ mode is more than 2.9 W, and the maximum power of LG_0,1_ is 2 .86W. The interference patterns corresponding to the four modes are shown at the bottom.

**Figure 4:**
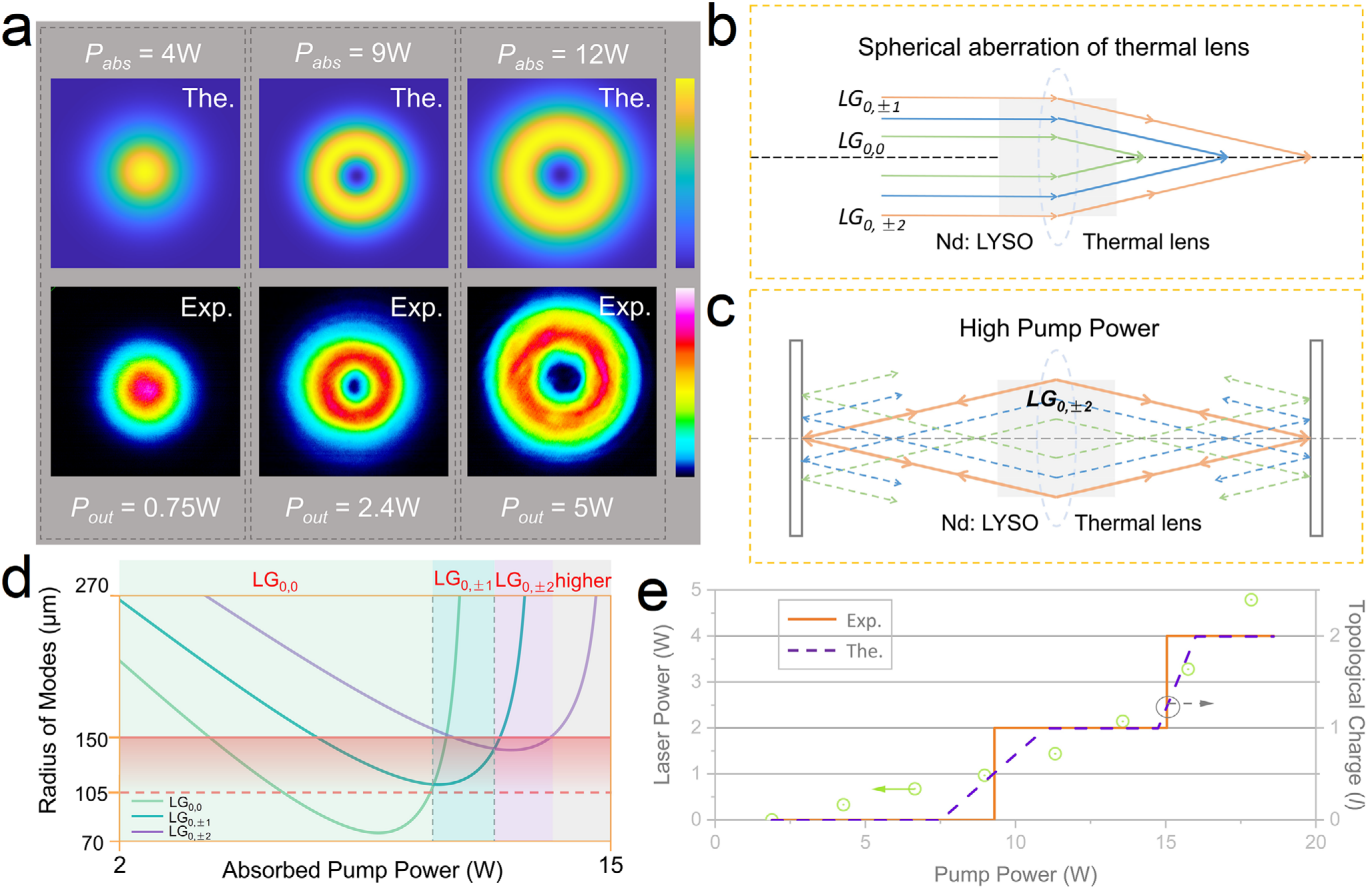
Dynamic evolutions of OAM states. a) Tunable OAM states from Nd:LYSO laser, i.e., OAM = 0, ‒1*ћ*, and ‒2*ћ* (from left to right). b) show the spherical aberration of the thermal lens. c) show the oscillating of LG_0, ‒2_ mode in cavity. d) Evolution of resonate modes in the cavity. The value 150 µm represents the radius of the pump beam, where 105 µm corresponds to 0.7 × 150. The analysis indicates that modes with pump‐beam radius ratios between 0.7 and 1 predominate in the laser operation. e) Comparison of the theoretical values and the experimental results. The purple line are the theoretically predicted modes, and the orange line are the experimental results.

The laser was operated in a continuous‐wave regime, where the output coupler was repeatedly adjusted to be perfectly parallel to the input mirror, but perpendicular to the optical axis, thus providing a high laser gain with mitigated asymmetry cavity losses. Moreover, the pump beam was perfectly parallel to the optical axis, eliminating the possibility of non‐collinear pumping and resulting in a high lasing efficiency. A Mach‐Zehnder interferometer was used to observe the topological charge of the OAM States (see Methods). **Figure** **3** shows the power handling capability and OAM characteristics of the Nd:LYSO laser, where three well‐defined OAM states are demonstrated, it can be seen the method of handedness selection based on the natural of crystal is effective since the clear interference patterns. As shown in Figure 3, the LG_0,0_ mode operated first in the low pump power with a maximum power close to 1 W, because of the perfect overlap of the fundamental mode with the pump light. After increasing the pump power, the LG_0,‒1_ mode operated with an output power of more than 2.5 W while the LG_0,0_ mode disappeared, and the LG_0,‒2_ mode operated at higher pump power with a slope efficiency of over 40% and a maximum power of 5.05 W, which thought to be caused by conical refraction, anisotropic gain, and thermal effects of the crystal (the power curves are shown in Figure S1, Supporting Information). The conical refraction of the fundamental mode created the OAM states component (‒1st order and 1st order), and thermal effects caused the OAM states to overlap with the pump light by changing the mode size, but the anisotropic gain of the crystal resulted in the amplification of only one handedness OAM states. The positive handedness OAM state were obtained by rotating the crystal 90 degrees about the *Y*‐axis to change the spatial distribution of the gain (see Figure S2, Supporting Information). Limited by the power of the pump light and the polarized absorption of the gain medium, higher topological charge OAM states were not produced, but the progression from 0 to ‒2 is consistent with the theoretical analysis (see **Figure** **4**a). The generated OAM states exhibit the following three features: quantized increment of the topological charge, handedness duality, and sequential number scaling of *l* (0 → −1 → −2), much similar to the generated new laser frequencies during the stimulated Raman scattering (SRS) process, i.e., a Raman‐like OAM states.

The generation of Raman‐like lasers is facilitated by a thermal lens‐assisted cavity design, we analyzed the thermal effects of the crystal to clarify the dynamics of OAM state tuning. The focal length of the thermal lens of a crystal can be expressed as:^[^ ^49^ ^]^

(6) $$f_{0}=\omega_{p}^{2}/2\cdot \frac{P_{th}}{4\pi K}\cdot \left[\frac{dn}{dT}+\left(n-1\right)\left(1+\upsilon \right)\alpha_{T}+2C_{r}n^{3}\alpha_{T}\right]$$

where *ω_p_* is the pump beam radius, *P_th_* is the thermogenic power, *K* is the thermal conductivity, $\frac{dn}{dT}$ is the thermo‐optic coefficient, *n* is the refractive index of the gain medium, υ is the Poisson's ratio, *α_T_* is the thermal expansion coefficient, *C_r_* is the photo‐elastic coefficient. Considering the spherical aberration of the thermal lens, the focal length equation can be expressed more precisely as:

(7) $$f_{T}=f_{0}\cdot \left[1.1-0.74\left(\frac{\omega_{l}}{\omega_{P}}\right)+1.22{\left(\frac{\omega_{l}}{\omega_{P}}\right)}^{2}\right]$$

where *ω_l_* is the radius of the laser modes (see Figure 4b).

The size of the LG modes can be expressed as $\omega_{p,l}=\omega_{0,0}\sqrt{2p+|l|+1}$, where *ω_p,l_* is the radius of LG*_p,l_*, *ω*_0,0_ is the radius of LG_0,0_, *p* is the radial quantum number, and *l* is the topological charge, we may note that the spatial distribution of different LG modes is separated. Figure 4c and Figure S3 (Supporting Information) show the schematic of transverse modes selection by using the thermal‐lens spherical aberration, i.e., controllable generation of desired OAM states. Specifically, the different thermal‐lens focal length will result in different stable zone of the laser cavity, thus leading to different resonant modes. Here, the thermal‐lens focal lengths at different power levels can be calculated according to Equation (7), and then the intracavity sizes of resonant modes can be simulated using ABCD matrix (see Figure S4, Supporting Information). Figure 4d shows the evolution of the different transverse modes with pump power, where LG_0,±1_ and LG_0,±2_ modes can be achieved in theory. A comparison of the theoretical calculations and experimental results is shown in Figure 4e, the topological charges are discretely increased both in experiment and theory as increasing the pump power. The LG_0,0_ mode would be first oscillated due to the lowest threshold with optimal spatial mode match with the pump beam. Then the thermal effect at the high power level will suppress the fundamental mode as the instability of the cavity, thus leading to the oscillation of LG_0,−1_ mode with a larger mode volume, in combination of the natural anisotropy of crystal gain for handedness selection. Similarly, a higher order OAM state (LG_0,−2_) can be produced for further power scaling with enhanced thermal effect.

## 3 Discussion

By comparing the performances of the OAM lasers using different samples with lengths of 8 and 13 mm, we found a positive correlation between crystal length and the purity of the OAM state (see Figures S5 and S6, Supporting Information), which was attributed to mode competition due to the overlap of the standing waves of two handedness in cavity, the weak gain at positions away from the focus amplifies the intensity difference between the two handedness. In addition, the output coupler is selected with an appropriate transmittance to achieve a balance between power handling and mode purity; low transmittance leads to simultaneous oscillation of both handedness due to ultra‐low intracavity losses (see Figure S7, Supporting Information), while too high transmittance reduces efficiency. Here, the three guidelines for generating Raman‐like OAM states are summarized as: i) low symmetry structure of the crystal offering anisotropic laser gain for opposite handedness to select the well‐defined OAM state; ii) suitable crystal length and optimized output coupler transmittance in such plane‐parallel laser cavity to enhance such separation between the different handedness; and iii) thermal‐lens effect assisted mode selection for the generation of Raman‐like OAM states.

In summary, by tailoring the OAM states through thermal‐induced mode competition and handedness selection with the natural anisotropy of crystal gain, a Raman‐like OAM laser with well‐defined helicity has been successfully demonstrated. Such OAM light source with compact structure and controllable states exhibits higher efficiency than the conventional bulk OAM laser and comparable efficiency as the novel single‐crystal fiber OAM lasers,^[^ ^50^, ^51^, ^52^, ^53^ ^]^ which can open new avenues for practical applications of high‐power vortex beams in various fields, such as precision micromachining of 3D microstructure,^[^ ^54^, ^55^ ^]^ holographic encryption,^[^ ^56^ ^]^ and optical metrology.^[^ ^57^ ^]^

## 4 Experimental Section

### Crystal Growth and Spectroscopy of Nd:LYSO Crystal

The Lutetium yttrium oxyorthosilicate (LYSO) host crystal is a solid solution of two different silicates, Lu_2_SiO_5_ and Y_2_SiO_5_, which is considered a promising matrix material for laser crystals, can grown by the traditional Czochralski technique. For the growth of high‐quality crystals, the raw materials need to be mixed well in proportion and subsequently pressed into tablets and heated, then, the polycrystalline material formed by the solid‐state reaction was loaded into an iridium crucible and a rectangular LYSO crystal was introduced as a seed thereby growing a high‐quality Nd:LYSO single crystal. The Nd:LYSO crystal crystallizes in a monoclinic structure and has the cell parameters: *a* = 1.2422 nm, *b* = 0.6670 nm, *c* = 1.0369 nm, *β* = 102.7854°, *V* = 0.8379 nm.^[^ ^58^ ^]^

The formula of the laser crystal used for the experiments is (Nd_0.005_Lu_0.4975_Y_0.4975_)_2_SiO_5_. As a low symmetry biaxial crystal, the absorption and fluorescence of Nd:LYSO have polarization properties. For the pump light whose electric field direction is parallel to the *X*‐axis of Nd:LYSO crystal (*E ∥ X*‐polarized), the absorption peak at 809 nm with an absorption cross section of 3.66 × 10^‒20^ cm^2^ while the absorption peak for the pump light *E ∥ Y*‐polarized is located at 815 nm with an absorption cross section of 1.371 × 10^‒20^ cm^2^, and for the *E ∥ Z*‐polarized pump light, the absorption peak at 810 nm with an absorption cross section of 2.761 × 10^‒20^ cm^2^. For the fluorescence of Nd:LYSO, the emission wavelength of *E ∥ X*‐polarized is 1079 nm, while 1076 nm of *E ∥ Y*‐polarized and *E ∥ Z*‐polarized at ^4^F_3/2_→^4^I_11/2_ transition.^[^ ^48^ ^]^

### Experiment Implementation and Interferometry

The complete experimental setup is shown schematically in Figure S8 (Supporting Information). In this step, a compact plane‐parallel cavity was used to perform the Raman‐like OAM states operation. After passing through a 4:3 telescope, the top‐hat‐shaped 808 nm pump beam was injected into a Nd:LYSO crystal with a spot radius of 150 µm in the focal plane (see Figure S9, Supporting Information). The bulk Nd:LYSO crystal with a cross section of 3 × 3 mm^2^, the end faces of which were polished but not coated with an anti‐reflective layer, and the direction of light transmission is parallel to the *Y*‐axis of the dielectric frame for the crystal. For the OAM states operation, the crystal was encapsulated in copper heat sinks and cooled by circulating water at 25 °C. The input mirror was coated with an anti‐reflective layer for 700–900 nm and a highly reflective coating for 1000–1100 nm, while the output coupler is partially transmittance in the 1000–1100 nm.

The topological charge measurement device consists of a Mach‐Zehnder interferometer and a beam profile camera. One arm of the Mach‐Zehnder interferometer has a delay line to acquire the interferogram and the corresponding phase information. As shown in Figure S8 (Supporting Information), the vortex beam is converted to a spherical wave by L3 (*f* = 50 mm), enabling co‐axis interference to produce spiral fringes. Since the wavefront curvature of the reference beam is smaller than that of the vortex beam, the counterclockwise spiral fringes represent negative OAM states.^[^ ^59^ ^]^

## Conflict of Interest

The authors declare no conflict of interest.

## Supporting information



Supporting Information

## Data Availability

The data that support the findings of this study are available in the supplementary material of this article.
