# Supplementary material for: Natural Crystal Structure for Generating Raman‐Like Orbital Angular Momentum States
Source: Adv Sci (Weinh). 2025 Apr 25;12(23):2500377. doi: 10.1002/advs.202500377 (PMC12199360; doi:10.1002/advs.202500377)
Supplement: Supplementary file 1 — Supporting Information [file ADVS-12-2500377-s001.docx]

*Supporting Information*

Natural crystal structure for generating Raman-like orbital angular momentum states

Tianxiang Meng, Changsheng Zheng, Yongguang Zhao*, Haohai Yu*, and Huaijin Zhang

T. X. Meng, C. S. Zheng, Y. G. Zhao, H. H. Yu, H. J. Zhang

State Key Laboratory of Crystal Materials and Institute of Crystal Materials, Shandong University, Jinan 250100, China

E–mails: [yongguangzhao@yeah.net](mailto:yongguangzhao@yeah.net)

[haohaiyu@sdu.edu.cn](mailto:haohaiyu@sdu.edu.cn)

**Section 1: Vortex beams, the orbital angular momentum of photon and Laguerre-Gaussian beams**

Vortex beams are those that have a wavefront that is distributed in a helical shape, and its expression of the light field in the cylindrical coordinate system contains the helical phase term exp(*ilφ*), which *l* is an arbitrary integer. The vortex beam is distributed in a hollow annular transverse field due to the uncertainty of the phase resulting in zero beam center intensity. In 1992, Allen et al. found that each photon in a vortex beam carries an orbital angular momentum (OAM) of ( is reduced to Planck's constant). Laguerre-Gaussian beams are near-axis approximate solutions of the Helmholtz equations in a cylindrical coordinate system, described by the product of the associated Laguerre polynomials and the Gaussian distribution function, are the eigenmodes of the column-symmetric cavity, with non-zero order Laguerre-Gaussian beams being the most common vortex beams. The Laguerre-Gaussian beams expression can be expressed as:

(1)

where *C_pl_* is a constant, *ω*_0_ is the radius of the fundamental mode, *l* is the angular quantum number, $p$is the radial quantum number, $L_{p}^{\left|l\right|}$ is the association Laguerre polynomial, $\omega(z)$and Φ can be expressed as:

$\omega(z)=\omega_{0}\sqrt{1+(\frac{z}{f}})^{2}$ (2)

$\Phi=(l+2p+1)\arctan\frac{z}{f}-k(z+\frac{r^{2}}{2R})$ (3)

where $k$is wave number and $R$is following as:

$R=z+\frac{f^{2}}{z}$ (4)

where *f* is Rayleigh length, which can be written as:

$f=\frac{\pi\omega_{0}^{2}}{\lambda}$ (5)

**Section 2: Characterization and the Power scaling of Raman-like OAM states**


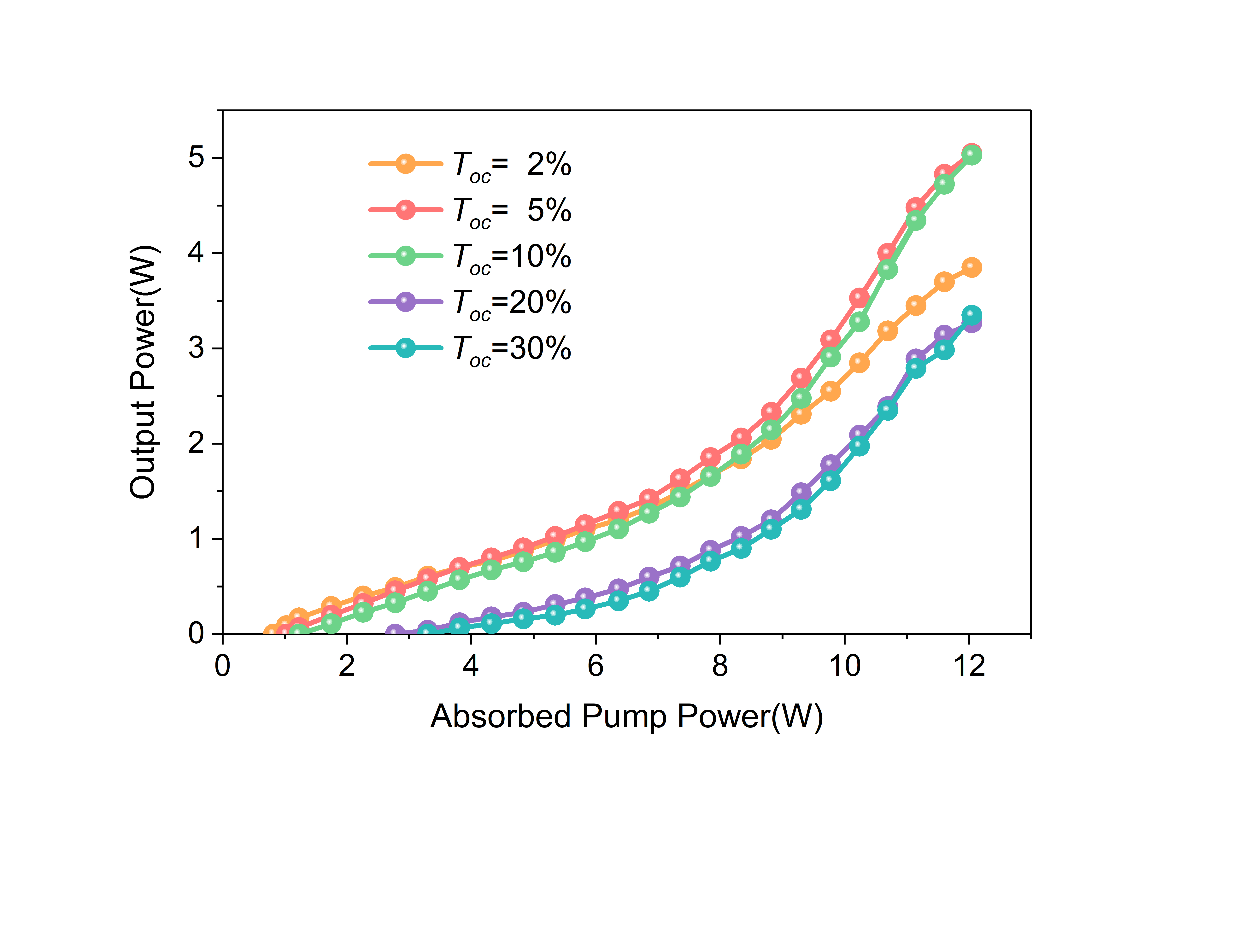


**Figure S1. Power handling capability of laser at different transmittance output couplers.**


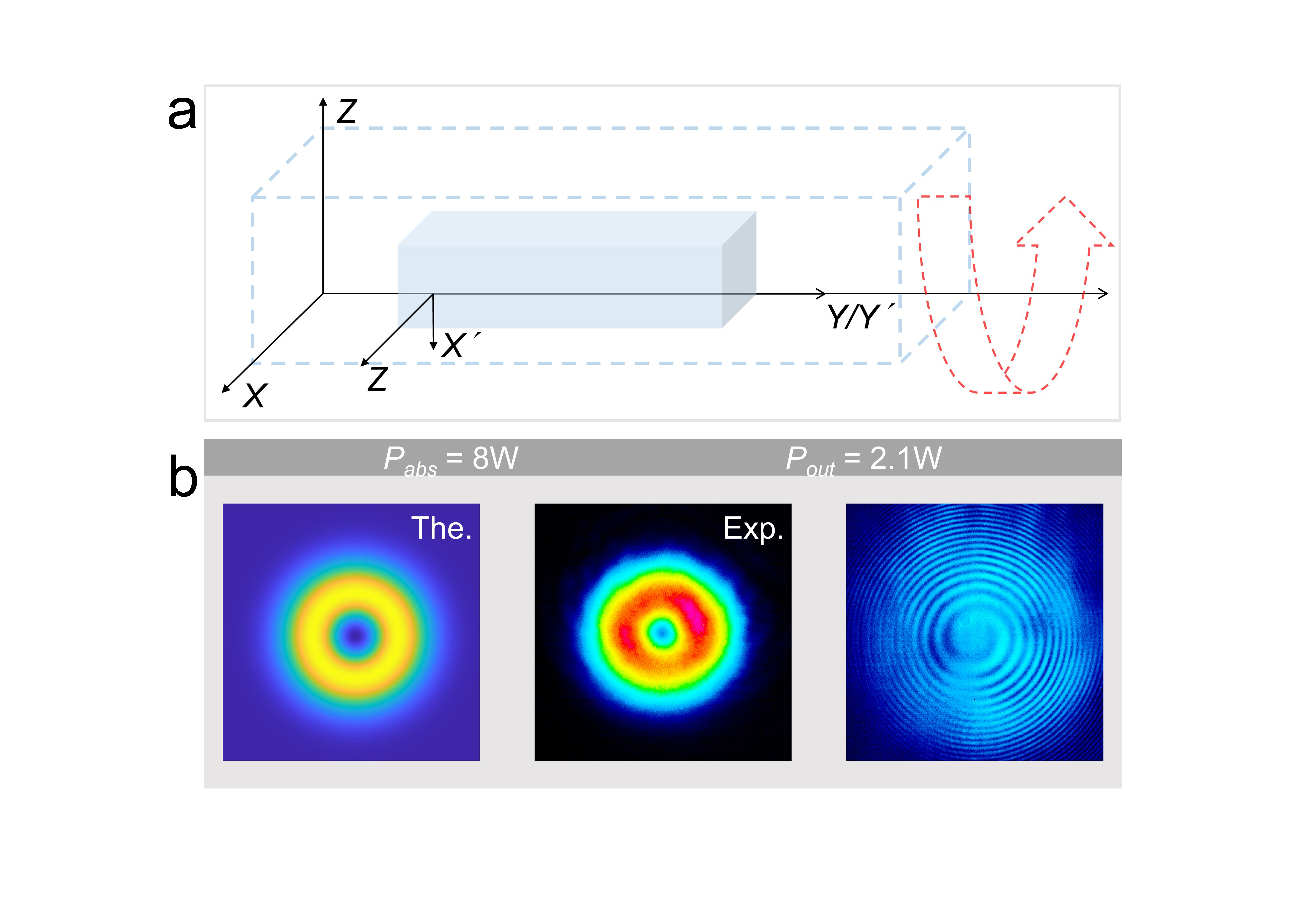


**Figure S2. Generation and measurement of the LG_0, +1_ mode. a** Schematic illustration of the crystal rotation process. Rotating the crystal by 90° around the optical axis with arrows indicating the rotation direction. Labels *X*, *Y*, and *Z* denote the three principal axes of the crystal before rotation, while *X’*, *Y’*, and *Z’* represent the principal axes after rotation. **b** Profiles and interference patterns of LG modes. The interference pattern with distinct fringes corresponds to a well-defined helical phase structure.

To simplify the calculations, thermal lensing effects due to thermal stress birefringence and crystal deformation are neglected, so the thermal lens focal length equation is rewritten as:

(6)

where *ω_p_* is the pump beam radius, *P_th_* is the thermogenic power, *K* is the thermal conductivity, $\frac{dn}{dT}$is the thermo-optic coefficient. For Nd:LYSO crystal, the thermal conductivity is ~ 3W/m*K* and the thermo-optic coefficient is ~ 9×10^−6^/*K.* Calculation of the sizes of the laser modes in cavity is based on the ABCD matrix, with all parameters identical to those of the experiment.


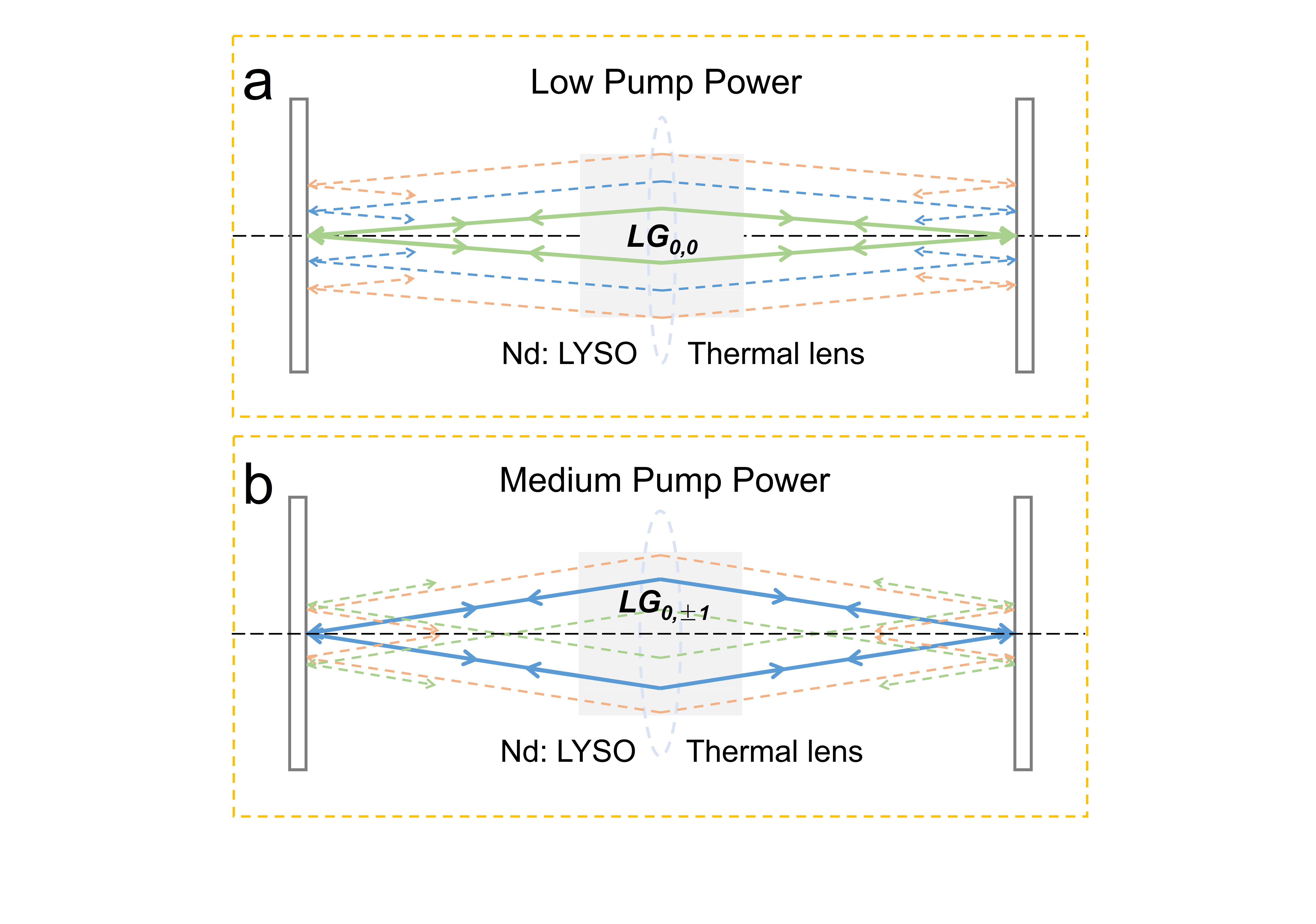


**Figure S3**. **Tuning of laser modes by pump power dependent thermal lens spherical aberration. a** LG_0,0_ mode dominance. At the low pump power, weak thermal lens effect induces negligible spherical aberration, allowing stable operation of the fundamental Gaussian mode (LG_0,0_). **b** LG_0, ±1_ mode excitation. Increasing the pump power to enhances thermal gradients, generating pronounced spherical aberration which selectively favors LG modes with azimuthal phase variation.

**

**

**Figure S4**. **Theoretical calculations.** Variation of the focal length of the thermal lens at three different power levels and variation of the mode sizes in the cavity with the thermal lens (inset).

**Section 3: Factors affecting of handedness selection methods based on natural properties of the gain medium**


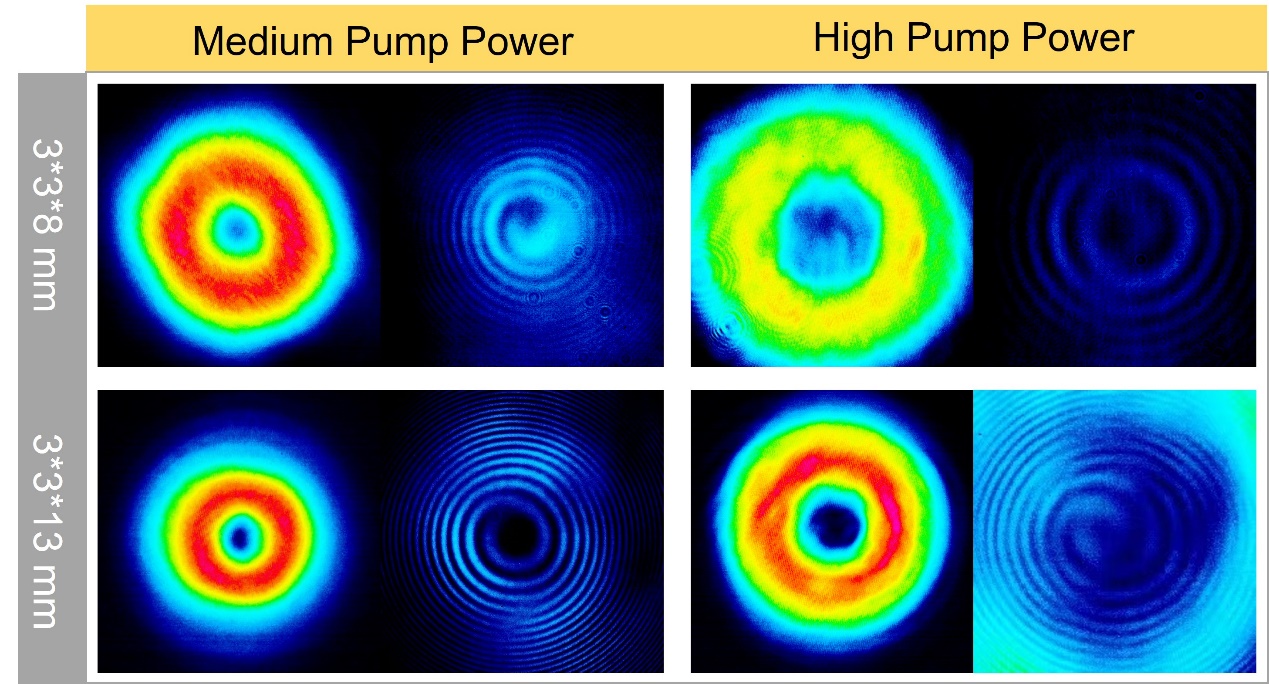


**Figure S5. The impact of crystal length on the purity of OAM modes.** OAM beams and the copresponding interference fringes obtained by using a 3×3×8 mm³ crystal as gain medium (up), unclear interference fringes indicate a disordered phase structure of the beam. The case with a 3×3×13 mm³ crystal (bottom), the high-contrast interference patterns indicate a well-defined handedness.


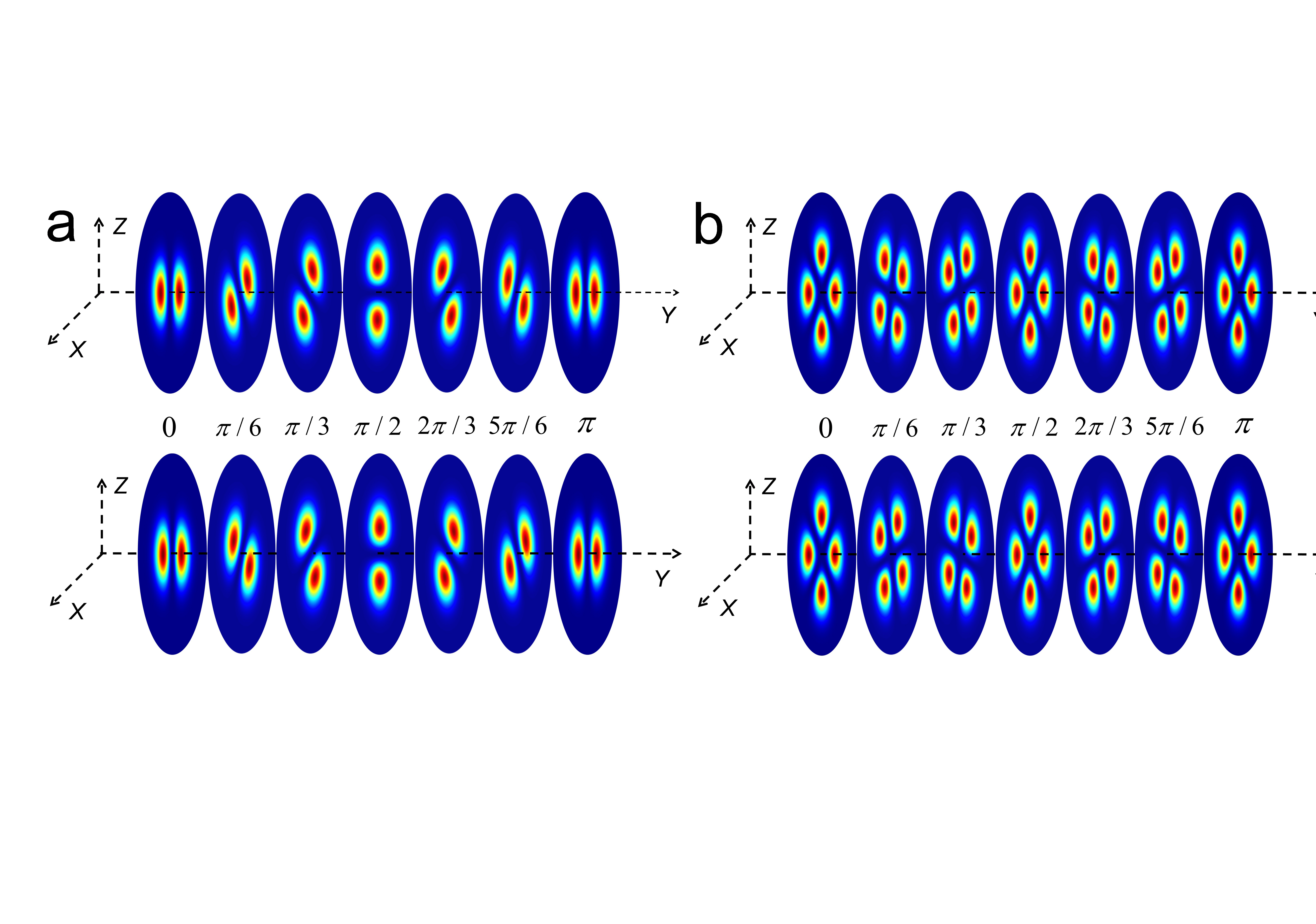


**Figure S6**. **Evolution of two handedness standing OAM waves in the cavity.** **a** LG_0,±1_ modes. **b** LG_0,±2_ modes.


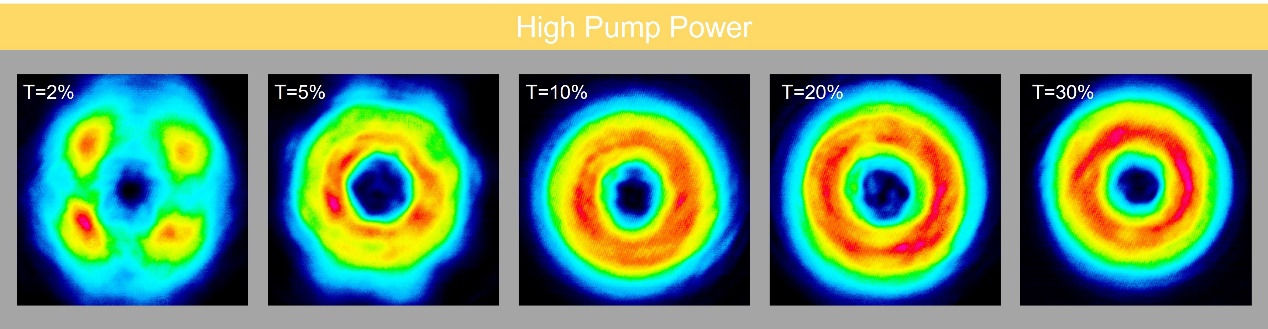


**Figure S7**. **Effects of output coupler transmittance on handedness selecting.** The OAM modes with less gain can operate when the output coupler has a low transmittance, resulting in a petal-like beam profile and the laser is an OAM degeneracy state.

**Section 4: Experimental demonstration of Raman-like OAM states**


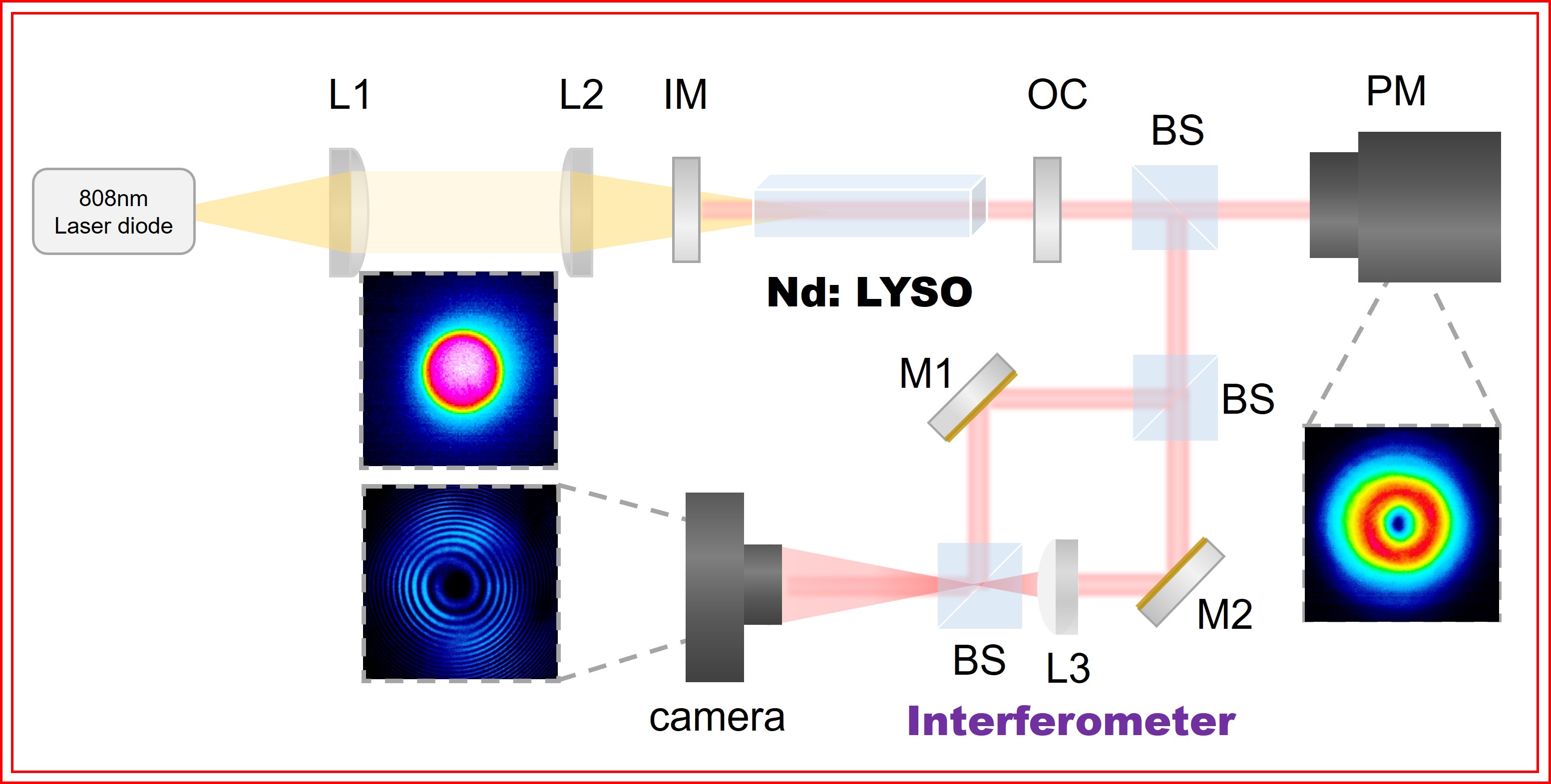


**Figure S8**. **The complete experimental setup.** Including a fiber-coupled 808 nm laser diode, focusing system, Fabry-Perot cavity, power meter, and order measurement device (Mach-Zehnder interferometer).


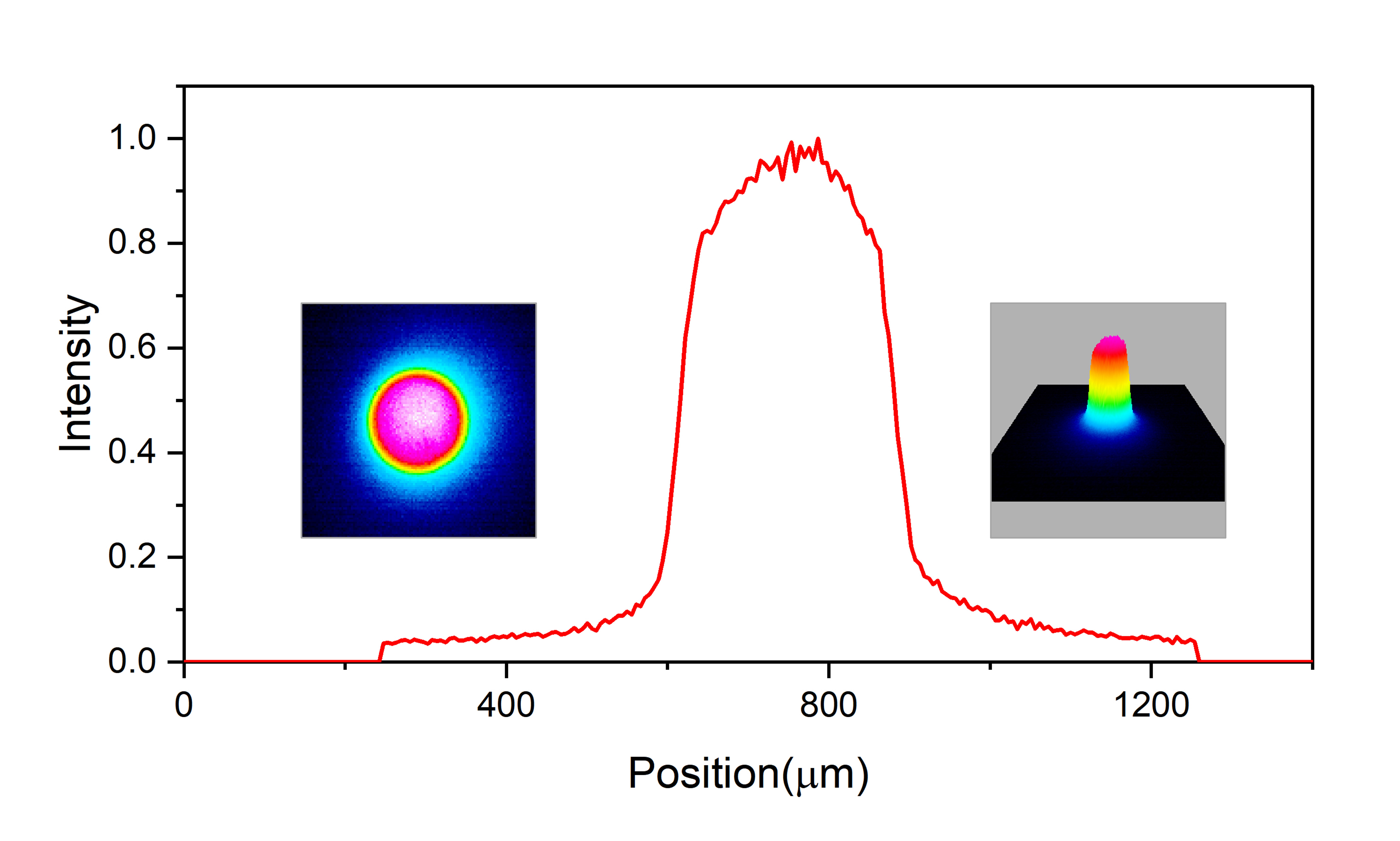


**Figure S9**. **Profile of the pump beam.** The pump beam after the focusing system is in the shape of a top-hat.
